# A Burden of Rare Copy Number Variants in Obsessive-Compulsive Disorder

**DOI:** 10.21203/rs.3.rs-3749504/v1

**Published:** 2024-01-03

**Authors:** Matthew Halvorsen, Elles de Schipper, Julia Boberg, Nora Strom, Kristen Hagen, Kerstin Lindblad-Toh, Elinor Karlsson, Nancy Pedersen, Cynthia Bulik, Bengt Fundín, Mikael Landén, Gerd Kvale, Bjarne Hansen, Jan Haavik, Manuel Mattheisen, Christian Rück, David Mataix-Cols, James Crowley

**Affiliations:** University of North Carolina at Chapel Hill; Humboldt-Universität zu Berlin; Broad Institute of MIT and Harvard and Uppsala University; Broad Institute of MIT and Harvard; Karolinska Institute; University of North Carolina at Chapel Hill; University of Oulu; Dalhousie University; Karolinska Institutet; Karolinska Institutet; UNC Chapel Hill

**Keywords:** Obsessive-Compulsive Disorder, OCD, copy number variant, CNV, genetic, genomic

## Abstract

Current genetic research on obsessive-compulsive disorder (OCD) supports contributions to risk specifically from common single nucleotide variants (SNVs), along with rare coding SNVs and small insertion-deletions (indels). The contribution to OCD risk from large, rare copy number variants (CNVs), however, has not been formally assessed at a similar scale. Here we describe an analysis of rare CNVs called from genotype array data in 2,248 deeply phenotyped OCD cases and 3,608 unaffected controls from Sweden and Norway. We found that in general cases carry an elevated burden of large (>30kb, at least 15 probes) CNVs (OR=1.12, P=1.77×10^−3^). The excess rate of these CNVs in cases versus controls was around 0.07 (95% CI 0.02–0.11, P=2.58×10^−3^). This signal was largely driven by CNVs overlapping protein-coding regions (OR=1.19, P=3.08×10^−4^), particularly deletions impacting loss-of-function intolerant genes (pLI>0.995, OR=4.12, P=2.54×10^−5^). We did not identify any specific locus where CNV burden was associated with OCD case status at genome-wide significance, but we noted non-random recurrence of CNV deletions in cases (permutation P = 2.60×10^−3^). In cases where sufficient clinical data were available (n=1612) we found that carriers of neurodevelopmental duplications were more likely to have comorbid autism (P<0.001), and that carriers of deletions overlapping neurodevelopmental genes had lower treatment response (P=0.02). The results demonstrate a contribution of large, rare CNVs to OCD risk, and suggest that studies of rare coding variation in OCD would have increased power to identify risk genes if this class of variation were incorporated into formal tests.

## INTRODUCTION

Obsessive-compulsive disorder (OCD) is a heritable complex neuropsychiatric condition characterized by persistent, intrusive thoughts and rituals. Current scientific literature supports a genetic contribution to OCD risk. Population-scale epidemiological studies indicate substantial familial clustering of the condition [1–3]. Based on twin study estimates, additive genetic factors account for 47% of variance in obsessive-compulsive symptoms [1]. Analyses of common genetic variation in OCD cases versus controls suggest that common risk variants explain around 28% of the phenotypic variance observed in the data [4].

Rare variant studies of OCD published in recent years have primarily utilized whole exome sequencing (WES) on trio cohorts (consisting of parents and an affected proband). Cappi *et al*. first described an analysis of WES data for 184 OCD trios and detected an excess of *de novo* (variant found in the proband, but absent in both parents) damaging coding single nucleotide variants (SNVs) and insertion-deletions (indels) in probands relative to unaffected trios, along with two genes recurrently hit with damaging mutations, *CHD8* and *SCUBE1* [5]. More recently, Halvorsen *et al*. described an analysis of 1,313 OCD cases, of which 587 were probands in complete trios [6]. In accordance with Cappi *et al*. they noted an excess of *de novo* damaging coding SNVs and indels in these OCD trios relative to unaffected trios. They also observed a general excess of protein-truncating SNVs and indels in singleton-cases relative to ancestry matched controls. They identified *CHD8* as a probable risk gene (Q < 0.3) in their analyses, which utilized summary statistics from Cappi *et al*., and identified an additional damaging coding de novo variant in their newly published trios [6]. It is critical to note that neither of these studies featured any comparison of copy number variants (CNVs). Since these variants are characterized by the deletion or duplication of thousands of bases, it is reasonable to hypothesize that in a sufficiently powered case/control comparison, given the excess of protein-truncating SNVs and indels already seen in OCD, there might be a similar excess of CNVs in OCD cases specifically impacting protein-coding genes.

There are CNV studies of OCD that have been published over the years, but these studies have small sample sizes, or do not specifically focus on OCD as a phenotype. The largest CNV study of OCD is from McGrath *et al* in 2014, as part of a joint study of OCD and Tourette Syndrome (TS) cases [7]. The study did not describe a general excess of large, rare CNVs, but did note that cases had an elevated rate of neurodevelopmental deletions relative to controls that was not statistically significant [7]. There have been other more recent CNV studies of OCD, but none featuring an exhaustive case/control comparison of CNV burden at a similar scale of McGrath *et al*. or the exome study described in Halvorsen *et al*. [8–10].

Given the excess of protein-truncating SNVs and indels already seen in OCD WES studies, we hypothesized that there might be a similar excess of CNVs in OCD cases, and that these CNVs specifically impact protein-coding genes. To test this hypothesis, we designed a case/control CNV study which benefits from usage of a more recently-developed genotype array platform (the Illumina Global Screening Array series; GSA), an ancestrally homogenous Scandinavian population ideal for genetic study, and rich clinical data available for enrolled cases.

## METHODS

### Samples

All OCD cases included in this study were collected in Sweden and Norway as part of the Nordic OCD and Related Disorders Consortium (NORDiC). The rationale, design and methods of the NORDiC study have been described previously [11]. In Sweden, the case-control arm of the study is referred to as NORDiC-SWE and all samples were collected across Sweden between 2015 and 2019. This study was approved by a local ethics board (Stockholm Regional EPN) and all participants provided informed consent. NORDiC-SWE OCD cases (63% female) have a primary International Classification of Disease, 10th revision [12] and/or Diagnostic and Statistical Manual of Mental Disorders, Fifth Edition [13] diagnosis of OCD from a multidisciplinary specialist OCD team (established with a semi-structured instrument such as the Mini-International Neuropsychiatric Interview [14] or the Structured Clinical Interview for DSM Disorders [15]. All patients were included in the study regardless of psychiatric comorbidity, as long as they fulfilled strict diagnostic criteria for OCD. Patients were excluded in cases of diagnostic uncertainty, such as OCD secondary to a neurological disorder or CNS insult, or where the differential diagnosis between OCD and an alternative condition was unclear. Swedish controls were sampled from LifeGene [16], a prospective population-based cohort of around 50,000 individuals in Sweden. Controls were unrelated to any OCD case to the third degree and unaffected with OCD based on self-report. Since the controls were inherited from those used for a GWAS of anorexia nervosa [17], potential controls were excluded if they had a lifetime history of anorexia nervosa and were largely female (~ 95%). Participants provided either blood or saliva for DNA extraction.

In Norway, the case-control arm of the study is referred to as NORDiC-NOR and all samples were collected across Norway between 2016 and 2019. This study was approved by the regional ethics board (REK West) and all participants provided informed consent. NORDiC-NOR OCD cases (65% female) had the same diagnostic process, and the inclusion and exclusion criteria as those in Sweden. Norwegian controls (50% female) were selected from NORMENT and were ages 18 to 65 at time of collection. They were screened for psychiatric illness via questionnaires, and included individuals have indicated that neither they nor any first-degree relatives have undergone any formal treatment. Participants provided either blood or saliva for DNA extraction. See [18] for more details.

### Array genotyping

The majority of samples in this study were genotyped on the Illumina GSA version 1, 2 or 3 (GSAv1, v2, v3), which include a common core of ~ 600,000 SNPs [19]. The one exception was the Norwegian controls, which were genotyped at deCODE Genetics using any array derived from GSAv1 that contained the same common core of SNPs [18]. The GSA samples were genotyped at LIFE&BRAIN in Bonn, Germany and the Norwegian controls were genotyped at deCODE Genetics in Reykjavík, Iceland. Some cohorts were genotyped in multiple waves and Table S1 provides the number of samples for each wave.

### Processing genotype array data

We obtained raw genotype array data (IDATs) for cases and controls, and processed them using the gtc2vcf pipeline (https://github.com/freeseek/gtc2vcf). We transformed IDATs into dataset-level VCFs with reference alleles listed relative to human reference build 37 (see Supplemental Methods for details), which simplified the dataset merge process. We merged all 10 input datasets on a subset of variants that have genotype missingness < 0.02 in each individual dataset. We identified a total of 542,466 variants that fit the full set of criteria. As a precaution, we took the subset of 537,278 variants that were non-ambiguous (not A/T or G/C), and were not indels for the merger. The raw merged dataset consisted of genotypes across these 537,278 variants for 2885 cases and 4227 controls.

### Quality Control (QC)

We carried out several rounds of sample-level QC using PLINK v1.90b4.9 to ensure that any case/control association results were not influenced by poor sample quality, sample swapping, cryptic relatedness or ancestry differences. See Supplemental Methods for a description of the full procedure carried out, and the number of samples removed at each step. After sample-level QC we were left with a total of 2325 cases and 3790 controls suitable for inclusion in a comparison of CNV burden between OCD cases and controls.

### CNV calling

We called CNVs on all NORDiC OCD cases and Swedish and Norwegian controls which we had sample-level Log R ratio (LRR) and B allele frequency (BAF) data for. All CNV calls were made on the same set of 537,278 variants common to all data described previously. The calling procedure utilized PennCNV v1.0.5 and QuantiSNP v2.2 to generate separate CNV callsets for each single sample, and then defined CNV loci based on the intersection of these callsets. See the Supplemental Methods section for a description of the full procedure.

### Sample-level QC specific to intensity metrics

Within each individual dataset, we performed outlier pruning on sample-level intensity metrics to remove poor-quality samples likely to have aberrant CNV call metrics. All metrics were computed by the PennCNV command ‘detect_cnv.pl’. Our dataset-level outlier pruning was focused on the standard deviation of the Log R Ratio (LRRSD), absolute value of waviness factor (absWF) and BAF drift. For each metric in a given dataset, a sample was marked as an outlier if it fell beyond 3 SDs of the mean. A sample was removed if it was an outlier for any of these metrics. After dataset-level pruning, all remaining samples had LRRSD ≤ 0.2, absWF ≤ 0.02, and BAF drift ≤ 0.001.

We next produced density plots of total CNV count per sample and total number of bases occupied by CNV per sample, and noted a small number of samples with unusually high counts that dataset-level QC failed to exclude. Based on visual inspection of the data we removed samples that had over 20MB of basepairs occupied by raw CNV calls, or a total number of separate raw CNV calls greater than 20.

### CNV filtering

The qualifying (≥ 15 probes, ≥ 30kb) CNV callset was put through a series of filter steps in order to produce a set of analysis-ready calls that are rare, and do not overlap loci naturally prone to copy number alterations. First, we removed CNVs that overlapped (here defined as > 30%) with loci 500kb from telomeres, or 500kb away from designated centromere loci. Next, we removed CNVs with 30% of bases overlapping “non-defined” (i.e., polyN) portions of the GRCh37 reference. We also removed CNVs overlapping previously reported and described segmental duplication loci [20]. Next, we removed CNVs where > 30% of bases overlapped loci from repeatmasker (downloaded 2021/06/21), corresponding to simple repeat, low complexity or satellite loci. CNVs that overlapped gene regions for T cell receptors or immunoglobulins were removed next. We removed loci where CNV calls were associated with samples from Epstein Barr Virus-transformed Lymphoblastoid Cell Lines [21], as utilized in Huang *et al*. 2018 [22]. Next, calls were subjected to a series of filters on CNV frequency. Calls were required to be found at < 1% frequency in gnomAD v2.1 non-neuro global and subpopulations. In addition, calls were required to be found at < 1% frequency in the full combined case/control dataset, along with < 1% frequency in each additional input dataset. The final step of our CNV filtering protocol utilized marker-level BAF and LRR values in a callsite for each given carrier sample, and used BAF metrics to validate or reject a call by determining if the distribution of BAF values is consistent with reported copy state, as described in https://biopsych.dk/iPsychCNV/ and utilized in [23] (see Supplemental Methods).

### Gene-based and breakpoint-based association tests

We used gene-based and breakpoint-based tests to determine if there were single genes or loci where overlapping CNVs were associated with OCD case status to a degree surviving multiple test correction, while also examining evidence of test statistic inflation. We defined four separate case/control groups for this analysis : 1) Swedish male, 2) Swedish female, 3) Norwegian male, and 4) Norwegian female. For each locus, we compared the proportion of cases with at least one rare, overlapping CNV to the proportion in controls, using a two-sided Cochran-Mantel-Haenszel exact test. To estimate genomic inflation, we used a case/control permutation-based approach described in [6]. Before formal tests we tested for genomic inflation of non-overlapping locus-based test statistics, and excluded CNVs overlapping 3 loci as a result (see supplemental methods).

We conducted gene-based tests of CNV burden in a manner described previously by others [24]. We tested deletions and duplications separately, and merged neighboring genes into single units if over 50% of overlapping CNVs impact both genes [24], leading to 988 separate tests in total. We used the Benjamini-Hochberg procedure to control for false discovery, considering results where false discovery rate (FDR) <0.05 as significant

We also constructed association tests based on CNV breakpoints. As with gene-based tests, we merged probes into separate units based on whether they are shared by over 50% of CNVs that impact them. We also used the same p-value adjustment procedure here as before. Once again, test statistics are well controlled for deletions (lambda = 1.02) and for duplications (lambda = 1.01).

### Global CNV burden tests

We tested for an association between case status and global CNV burden using linear and logistic regression models, with covariates. We first constructed a null model for the total count of raw CNVs per sample. Without additional covariates, we saw that case status was associated with the raw number of CNV calls made in a given sample (estimate=−0.17, P = 0.04). For our null model, we selected major principal components (PCs 1–5) and sex. Notably, PC5 is a clear predictor of Swedish versus Norwegian ancestry, in a manner that is not dataset-specific (see Supplemental Fig. 2). We considered additional covariates (PCs 6–20, LRRSD) and added them to the model if they were associated with both raw CNV count and with case status. We found PC 7 and LRRSD to be associated with both, and added them to the model. The logistic regression model in global comparisons of burden was:

OCD_case ~ PC1 + PC2 + PC3 + PC4 + PC5 + PC7 + SEX + LRR_SD + burden_metric

A linear regression model used these same covariates, but here instead the so-called ‘burden metric’ was the outcome and OCD case status was the critical predictor.

## RESULTS

### Case/Control Cohort

**Figure 1:**
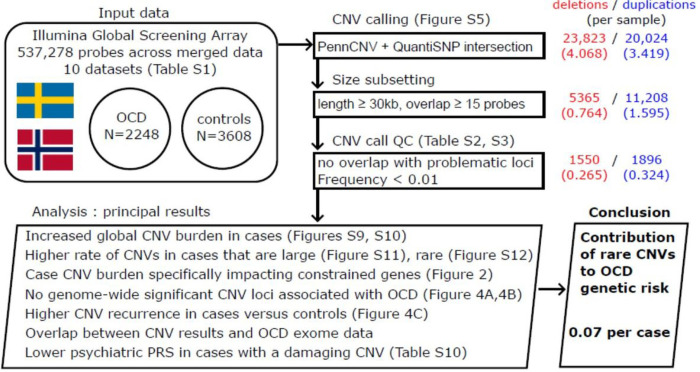
An overview of the study design and principal results from the analyses carried out. We have constructed a CNV case/control study using samples from Illumina GSA and its derivatives. We use extensive CNV call quality control to bring our CNV call rate down to around 0.6 per sample. Our principal results all point to a contribution to OCD genetic risk from large, rare CNVs, at a rate per sample of around 0.07 (95% CI 0.02–0.11, P=2.58×10^−3^).

The raw case/control cohort consisted of 2,885 cases and 4,227 controls, spread across 10 datasets (Fig. 1, Supplemental Table 1). All samples were genotyped on versions or derivatives of the Illumina GSA (Supplemental Table 1). We found a total of 537,278 probes shared across all datasets within this study, and utilized these probes for both quality control and CNV calling.

We isolated a subset of the cohort that were of high technical quality and were suitable for case/control comparisons (see Methods for details). A total of 2,248 cases and 3,608 controls were included in our formal CNV analyses (Fig. 1, Supplemental Table 1). We did not see evidence of stratification of OCD case status across any of the major principal components (Supplemental Fig. 1), though it was clear that Swedish and Norwegian ancestry did separate across these components and should be accounted for (Supplemental Fig. 2). Most of the variance within the data was explained by the first 5 PCs (Supplemental Fig. 3).

### CNV calling and filtering

We called and analyzed large CNVs (≥ 15 probes, ≥ 30kb). A large number of these calls were present in sample-level data (1.42 calls per sample, Supplemental Tables 2–4). We retained CNVs outside of genomic loci prone to noisy intensity values, and were found at a frequency < 0.01 in the cohort as well as the gnomAD v2.1 structural variant callset (see Methods). This procedure led to a higher degree of comparability across separate datasets (0.59 calls per sample, Supplemental Tables 2–4).

We compared cases and controls for evidence of systemic differences in raw CNV call count, LRRSD and filtered CNV call count. LRRSD metrics across datasets indicated that the data were of good quality. Looking at ANGI controls, which underwent clustering before we received the data, the mean LRRSD metrics were higher, but still in range of other included datasets (Supplemental Fig. 4). As indicated before, raw CNV call counts had appreciable differences between datasets, while QC-pass CNV call counts were well-harmonized across the data (Supplemental Figs. 5 and 6).

Out of an abundance of caution, we compared the global burden of small CNVs in cases versus controls to determine if there are clusters of calls that pile up in a manner suggestive of batch effect. We specifically noted an elevation of small (30kb-100kb) CNV deletion signal (lambda = 1.21), which was driven exclusively by 19 small CNV calls clustering around 3 loci (see Methods). We excluded these small CNVs from further analyses, eliminating genomic inflation for small deletions (lambda = 0.98, Supplemental Fig. 7). No similar clustering that was suggestive of batch effect was present in CNVs calls over 100kb in size (Supplemental Fig. 8).

### Global CNV burden

We found that OCD cases had an excess burden of large (> 30kb) rare CNVs relative to unaffected controls (OR = 1.12, P = 1.77×10^−3^). More of this excess burden appears to come from deletions (OR = 1.16, P = 8.41×10^−3^) than from duplications (OR = 1.09, P = 0.06, Supplemental Fig. 9). Leave-one-out analyses showed that these results were not driven by one input dataset (Supplementary Fig. 9), or by one covariate (Supplementary Fig. 10). Every additional basepair of large deletion made a sample more likely to be a case (OR = 1.047 per 100kb, P = 2.31×10^−3^), along with every additional basepair of duplication (OR = 1.033 per 100kb, P = 1.81×10^−3^). Consistent with this, OCD cases carried an excess burden of large (> 1MB) CNVs (OR = 2.01, P = 3.35×10^−4^, Supplementary Fig. 11). Ultrarare CNVs found only once in the case/control cohort conferred greater relative risk for OCD (OR = 1.21, P = 1.30×10^−3^, Supplementary Fig. 12), consistent with particularly penetrant, risk-conferring CNVs being subject to purifying selection. This singleton CNV signal was driven mainly by deletions (Supplementary Fig. 13).

### CNV burden is concentrated in protein-coding regions

OCD cases were more likely to carry CNVs that impact protein-coding regions of the genome (OR = 1.19, P = 3.07×10^−4^). There was no evidence for a case burden relative to controls for CNVs not overlapping any protein-coding bases (OR = 1.04, P = 0.50). Consistent with the burden of large CNVs in cases, the accumulation of CNV-impacted protein-coding genes increased OCD case risk (OR = 1.07 and P = 1.99×10^−3^ for each deletion-impacted gene, OR = 1.04 and P = 3.48×10^−3^ for each duplication-impacted gene).

**Figure 2:**
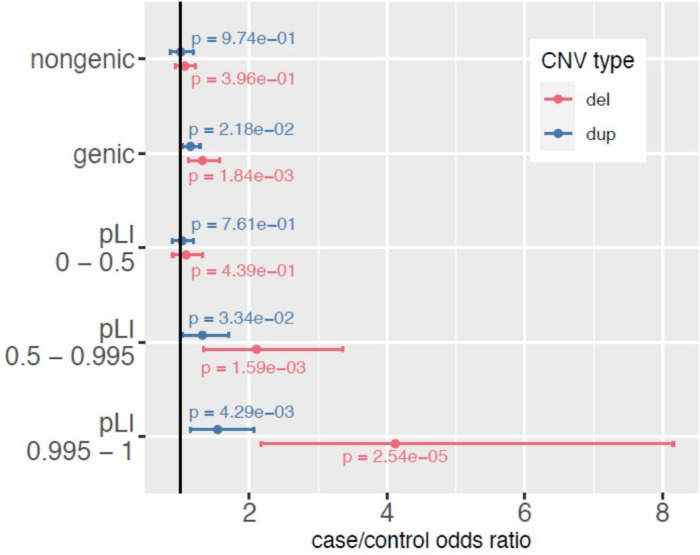
CNV burden (deletions, duplications) partitioned by overlap with protein-coding genes. The odds ratio estimate for case status for each additional CNV (deletions in red, duplications in blue) is depicted with a dot while the 95% confidence interval for the estimate is depicted with bars. There is no evidence for case risk being conferred by CNVs that don’t overlap a protein-coding base (P>0.05 for both deletions and duplications). CNVs that confer OCD risk instead appear to overlap protein-coding regions, specifically those that code for genes that are loss-of-function intolerant (pLI>0.5).

Case CNV signal was concentrated within genes that are dosage sensitive. Cases carried an excess of CNVs that overlap at least one protein-coding gene that is more likely to be intolerant to loss-of-function (pLI > 0.5, OR = 1.60, P = 6.37×10^−8^, Fig. 2). There was no difference in burden of CNVs that do not carry at least one of these genes (OR = 1.04, P = 0.48). We also utilized recently described [25] sets of data-derived haplosensitive and triplosensitive genes and found that CNV burden was elevated in both sets (Supplemental Fig. 13).

### CNV burden impacted evolutionarily constrained bases

**Figure 3:**
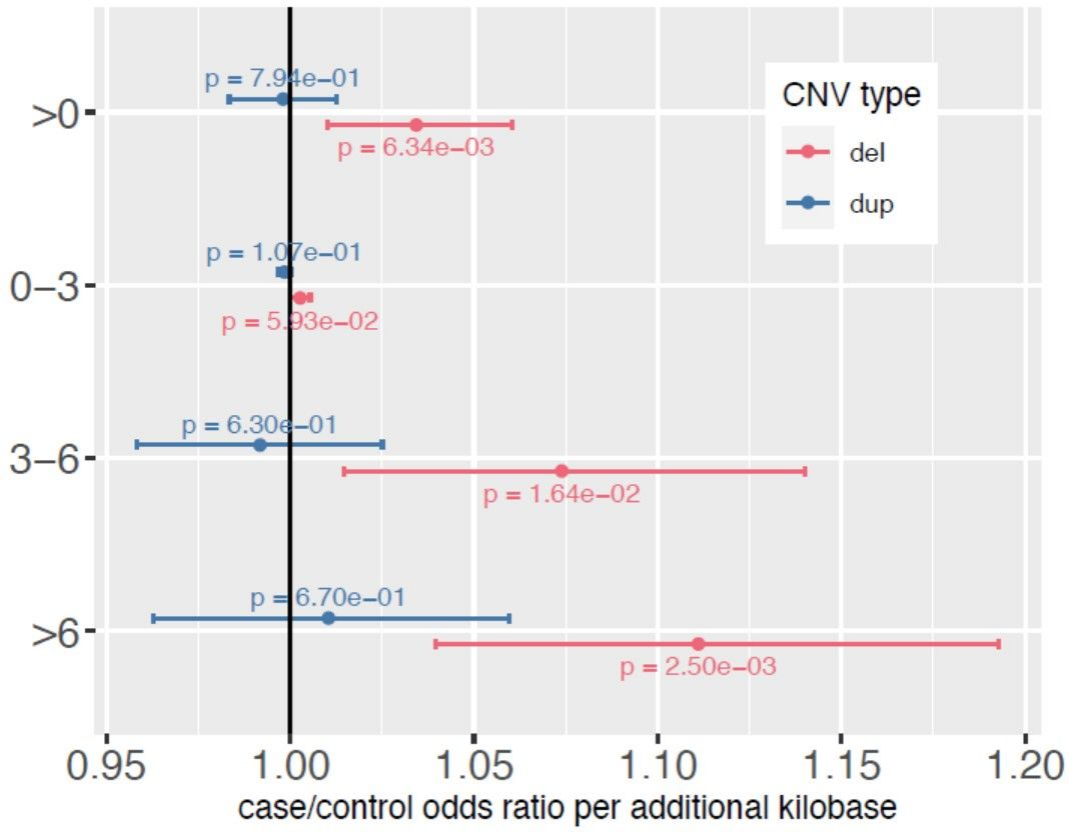
Number of bases impacted by CNVs (deletions, duplications) partitioned by mammalian constraint score. The odds ratio estimate for case status for each additional kilobase impacted by CNVs (deletions in red, duplications in blue) is depicted with a dot while the 95% confidence interval for the estimate is depicted with bars. In general, each kilobase of DNA that is deleted increases OCD risk, in a manner where the risk conferred increases when the bases deleted are more constrained. This effect is not observed for duplications.

OCD cases had a higher number of evolutionarily constrained bases impacted by CNV deletions than controls (OR = 1.03 per kbp, P = 6.34×10^−3^). There was no significant case/control difference in the number of constrained bases impacted by duplications (OR = 0.998 per kbp, P = 0.79). We found that CNV deletion burden preferentially loads onto bases with particularly high phyloP scores (Fig. 3), consistent with deletions impacting genomic loci that are intolerant to variation. Repeating this test on CNVs that did not impact a coding base, we did not note any significant case/control difference in constrained bases burdened by CNVs (Supplemental Fig. 14).

### Gene-based tests of CNV burden

**Figure 4:**
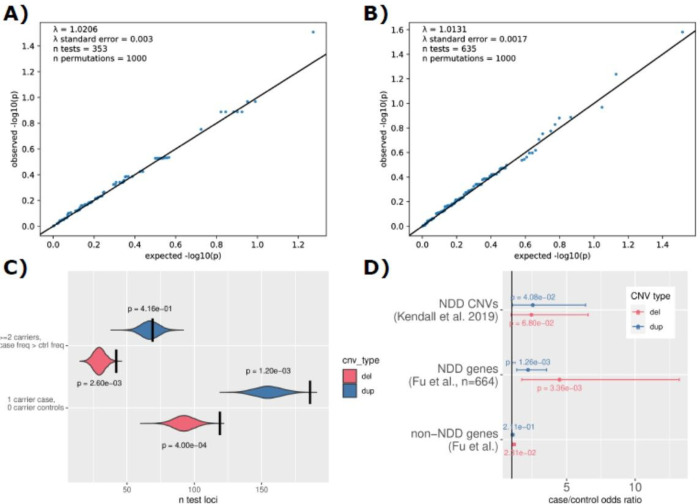
Lack of genome-significant CNV impacted loci in OCD cases versus controls likely due to low power. **A)** QQ plot for clumped gene-based test results specific for deletions. **B)** QQ plot for clumped gene-based test results specific for duplications. **C)** Results from permutation tests of CNV burden in recurrence in OCD cases relative to controls. **D)** Tests of association between neurodevelopmental disorder (NDD) CNV burden and OCD case status. Odds ratio for each additional CNV (deletions in red, duplications in blue) is depicted with a dot while the 95% confidence interval for the estimate is depicted with bars. All detectable duplication excess in cases appears to impact neurodevelopmental genes (n=664, from Fu *et al*. 2022, [27]) while the deletion excess in cases appears to impact both neurodevelopmental genes and unknown genes outside of this geneset.

We failed to identify any test statistics where the level of significance passed the threshold for significance (988 tests, FDR-adjusted P < 0.05). There was no evidence of genomic inflation within deletion test statistics or duplication test statistics (lambda = 1.02 and lambda = 1.01, respectively, Fig. 4A and 4B). In spite of no individual loci being implicated, the overall CNV burden described in OCD cases suggests that a larger cohort size is likely to provide the sufficient power required. In particular, cases were more likely to have CNVs where only one sample overlaps the affected area, and specifically, cases have an elevation of loci impacted by at least two deletions beyond what case/control permutation predicts (Fig. 4C). Summary statistics from these tests have been included (Supplemental Table 6), along with statistics from breakpoint-based tests (Supplemental Table 7).

### Burden of neurodevelopmental CNVs

In general, we found that OCD cases carried a higher burden of neurodevelopmental CNVs than controls. Burden of neurodevelopmental CNVs as defined in Kendall *et al*. [26] increased OCD case risk (OR = 2.49, P = 6.04×10^−3^), as did burden within specific genes implicated with neurodevelopmental disorders from Fu *et al*. [27] (n = 664, OR = 2.54, P = 1.91×10^−5^). Although the deletion contribution to this result was higher, there was a discernible contribution from duplications as well (Fig. 4D).

### Overlap with exome sequencing studies of OCD

We found non-random overlap between genes impacted by case-only single-gene CNVs in our study and prior OCD exome study statistics from Table S15 of [6]. We took a set of genes that were impacted by at least one single-gene case CNV and no single-gene control CNV (n = 149 genes). These genes had an elevated count of loss-of-function and damaging missense *de novo* mutations across 771 trios (observed = 9, expected = 3.94, one-sided poisson P = 0.02) and an elevated count of loss-of-function variants in 476 cases versus 1,761 controls (observed = 26, expected = 17.30, one-sided poisson P = 0.03). We set up a Transmission and De Novo Association (TADA) analysis using the same methods described previously [6] and the summary statistics from Table S15, with NORDiC case/control count statistics added. No genes beyond the already-described *CHD8* passed the threshold of Q < 0.3 for being classified as a probable risk gene (Supplemental Table 8), though the gene that comes closest, *ZMYM2* (Q = 0.32), has been implicated in neuropsychiatric phenotypes across multiple publications [27–29].

### OCD polygenic risk in deleterious CNV carriers

We hypothesized that individuals carrying deleterious (pLI > 0.995, neurodevelopmental as in Kendall *et al*., or neurodevelopmental as in Fu *et al*.) CNVs were more likely to have lower neuropsychiatric polygenic risk. This would be consistent with higher-powered studies of other neuropsychiatric conditions [30]. To test this, we utilized polygenic risk scores (PRS) computed from three different GWAS summary statistics: standing height (Pan-UKB, https://pan.ukbb.broadinstitute.org) (N = 360,388, as a negative control), OCD [4] (2,688 cases, 7,037 controls), and a cross-disorder study of psychiatric conditions [31] (162,151 cases, 276,846 controls). We tested for an association between deleterious CNV burden and normalized PRS, using the same covariates as those in global CNV burden analyses, and performing separate tests for deletions and duplications.

Of the six tests we performed (Supplemental Table 9), we identified one significant (p < 0.05) association, between deleterious CNV deletions and cross-psychiatric condition study PRS. In this comparison, deleterious CNV deletion carriers in our case cohort had lower normalized psychiatric PRS than non-carriers (estimate=−0.45, P = 3.35×10^−3^). While this PRS is not OCD-specific, the summary statistics underlying it do include OCD cases, and given how much larger the sample size is, it likely captures pleiotropic common risk variants that increase risk for multiple psychiatric conditions at once.

### Clinical features of carriers of deleterious CNVs

We performed an analysis of clinical features of case carriers of these deleterious CNVs versus non-carrier cases (see Supplemental Table S10 for carrier status per sample). We focused on the Swedish subset of the case cohort (n = 1612) where we had access to detailed clinical information on each participant.

We first explored the association between deleterious deletions and duplications and the presence of key psychiatric comorbidities (ASD, ADHD, TS/chronic tic disorder, schizophrenia, and bipolar disorder) through contingency tables and Chi-Square statistics (or Fisher exact tests, when relevant). We found that 6 (4.1%) of the 147 individuals with comorbid ASD had neurodevelopmental duplications, compared to 6 (0.4%) of 1,465 individuals without comorbid ASD (Chi-square = 24,3, df = 1, P < 0.001). The remaining psychiatric disorders were not significantly associated with neurodevelopmental duplications. No significant associations emerged for neurodevelopmental deletions.

We further explored if the presence of duplications or deletions was associated with treatment outcomes in a sub-cohort of Swedish individuals with complete treatment data (n = 846). We found that individuals with deletions (but not duplications) in specific neurodevelopmental disorder genes improved on average 16% on the YBOCS, whereas individuals without such deletions improved 47% on the YBOCS, a statistically significant difference (independent samples t-test; t=−3.03, df = 854, 2-sided P = 0.02).

## DISCUSSION

We have compiled what, to our knowledge, is currently the largest OCD case/control study of rare CNVs, and our results support a contribution of these variants to OCD genetic risk. This contribution came specifically from rare CNVs that overlap protein-coding regions of the genome, as there was no detectable difference in noncoding CNV burden between cases and controls. Large, ultra-rare CNVs appeared to confer the highest amount of OCD relative risk. Even when controlling for the total number of CNV-impacted bases, OCD cases had a higher number of deleted bases that are under high mammalian evolutionary constraint. In a manner consistent with OCD WES studies, coding region CNVs impacting loss-of-function intolerant protein-coding genes appear to confer more substantial OCD risk than those that do not. There was no single locus in the genome where CNV burden predicted OCD case status at a level that survived multiple test correction. The distribution of case CNV calls in the genome was non-random, and consistent with a pattern in which distinct CNV risk loci exist, but we have insufficient sample size to be able to detect them.

Our study benefited from the uniquely rich clinical information that is available from the participants in the NORDiC study. In particular, we established a specific association between neurodevelopmental duplications and ASD (not other comorbidities), although no significant associations emerged for neurodevelopmental deletions. The results suggest that whereas neurodevelopmental duplications in OCD can be, at least in part, explained by the presence of comorbid ASD, our findings regarding deletions appear to be independent of key psychiatric comorbidities. We also found a tentative association between deletions and multimodal treatment response in a sub-cohort of individuals who had treatment outcome data, whereby individuals with deletions in neurodevelopmental disorder genes were less likely to respond to treatment. However, these results should be interpreted with caution because this analysis only included 8 cases with deletions in neurodevelopmental disorder genes. Larger samples are needed to confirm this finding.

The case/control CNV callset here and the previously published gene-based WES summary statistics from Halvorsen *et al*. 2021 overlap in a nonrandom manner consistent with the presence of multiple OCD risk genes impacted in both datasets [6]. This indicates that the process of calling CNVs from WES data and forming gene-based summary statistics that incorporate SNV, indel and CNV calls is a worthwhile endeavor. Consistent with this, recent large WES analyses have benefited greatly from incorporating CNV call information into gene based tests, and methods for making CNV calls from WES data and incorporating them into analyses have been optimized [27, 32]. CNV burden and damaging SNV/indel burden, in a scenario where sample size is sufficiently large, should point to a consistent core set of risk genes where damaging coding variation substantially increases risk of OCD.

## CONSORTIA

### Members of the Nordic OCD & Related Disorders Consortium (NORDiC):

Julia Boberg, Long Long Chen, James J. Crowley, Elles de Schipper, Diana R. Djurfeldt, Jan Haavik, Kristen Hagen, Matthew W. Halvorsen, Bjarne Hansen, Kira D. Höffler, Anna K. Kähler, Elinor K. Karlsson, Gerd Kvale, Paul Lichtenstein, Kerstin Lindblad-Toh, Manuel Mattheisen, David Mataix-Cols, Kathleen Morrill, Hyun Ji Noh, Christian Rück, Thorstein Olsen Eide, Nora I. Strom, Tetyana Zayats
